# mRNA expression and localization of bNOS, eNOS and iNOS in human cervix at preterm and term labour

**DOI:** 10.1186/1477-7827-3-33

**Published:** 2005-08-10

**Authors:** Susanne Abelin Törnblom, Holger Maul, Aurelija Klimaviciute, Robert E Garfield, Birgitta Byström, Anders Malmström, Gunvor Ekman-Ordeberg

**Affiliations:** 1 Department of Woman and Child Health, Division of Obstetrics and Gynaecology, Karolinska University Hospital, Karolinska Institute, 171 76 Stockholm, Sweden; 2 Department of Obstetrics and Gynaecology, Division of Reproductive Sciences, University of Texas Medical Branch, Galveston, Texas, TX 77555-1062, USA; 3 Department of Obstetrics and Gynaecology, University of Heidelberg, 69115 Heidelberg, Germany; 4 Dept of Experimental Medical Science, BMC, University of Lund, 221 84 Lund, Sweden

## Abstract

**Background:**

Preterm birth is the primary cause of the neonatal mortality and morbidity. There will be no preterm birth without a cervical softening. Nitric oxide (NO) is shown to be a mediator of term cervical ripening. The aim of this study was to investigate mRNA expression of the three isomers of NO synthases (NOS) and to identify them by immunohistochemistry in the human cervix at preterm birth compared to term.

**Methods:**

The three isomers of NOS- inducible (iNOS), endothelial (eNOS) and neuronal (bNOS) – were investigated in the human cervix. The expression of mRNA was determined using Real-Time Multiplex RT-PCR. The localisation of synthases in the cervical tissue was analysed using immunohistochemistry. Cervical biopsies were obtained from 4 groups of women without clinical signs of infection: preterm (PTL), term labour (TL), preterm not in labour (PTnotL) and term not in labour (TnotL) patients. One-Way ANOVA, Kruskal-Wallis, Student t-test or Mann-Whitney test were applied as appropriate to determine statistically significant differences among the groups.

**Results:**

Patients in preterm labour had significantly (p < 0.01) higher mRNA levels of all the three NOS isomers compared to those in term labour. Women not in labour, irrespective of gestational age, thus with unripe cervices, had significantly lower eNOS mRNA levels compared to those in labour (p < 0.01). Immunoreactivity for all three NO synthases was observed in each examined sample in all groups. The bNOS staining was the most prominent.

**Conclusion:**

The mRNA levels were higher in the preterm labour group compared to the women at term labour. The significant increase of the eNOS mRNA expression, from the unripe to the favourable cervical state during labour, may indicate a role of eNOS and supports the role of NO in the cervical ripening process. All the three synthases were identified by immunohistochemistry in all the groups of study.

## Background

The frequency of premature birth has not changed significantly during the past two decades and the basic mechanisms underlying the initiation of both preterm and term cervical ripening and labour remain unknown [1]. No effective treatment for preterm labour (PTL) that reduces perinatal mortality and inhibits preterm delivery exists [2]. To accomplish a preterm delivery the myometrial contractions must be coordinated with a preterm ripening of the cervix. The cervical remodelling detected clinically, consists of softening, effacement and dilation. This corresponds to vast changes in the extracellular matrix (ECM) composition and tissue remodelling by proteolytic enzymes [3,4]. The cervical ripening procedure is to be considered an inflammatory reaction. The 100-fold increase of interleukines is likely to be responsible for the recruitment of leukocytes/influx of white blood cells triggering the inflammatory process [1,5,6].

Many studies have revealed the importance of nitric oxide (NO) as a major mediator in numerous biological processes in the human. NO controls the key elements that enable reproduction and NO production is essential for maintaining pregnancy [7]. It is synthesized by any of the three NO synthases (NOS)- inducible (iNOS), endothelial (eNOS) and neuronal (bNOS)- from the amino acid L-arginine and oxygen to equal amounts of NO and citrulline [8]. Two of the NOS enzymes, bNOS and eNOS, are constitutively expressed and calcium dependent. The third enzyme, iNOS, is calcium independent and can be induced by various cytokines. iNOS synthesizes NO in large quantities during a long period of time [9]. bNOS and eNOS occur in smaller amounts than the iNOS.

The role of NO as an inflammatory mediator in cervical ripening is complex but seems to be important – NO has been proposed to act as the final effector in this process [10]. The results from earlier studies are conflicting, but all three NO synthases have been identified in the human cervix at term pregnancy [11-14]. NO amplifies the cytokine cascade in acute inflammatory response [15]. Together with PGE_2 _and PGI it is the most potent vasodilator and stimulates the matrix metalloproteinases (MMPs) activity in the cervical tissue [15,16]. In 1997 Chwalisz and Garfield raised the theory that NO induced local vascular permeability by leukocyte infiltration, activating MMPs and also modulating the proteoglycan synthesis during the cervical remodelling [17]. Locally administered NO-donors induce cervical ripening in humans [18]. They have been found to enhance the COX-2 enzyme activity which thereby up-regulates the prostaglandin synthesis and leads to increased releases of PgE_2 _and PgF_2α _[19]. Prostaglandins may be the mediators of NO-driven cervical ripening and dilation [1]. The cervical bNOS protein expression has been shown to be significantly higher at term labour (TL) compared to term not in labour [12,14]. These data contradict the study by Ledingham et al. that could not show any increase of the bNOS protein expression following the onset of labour compared to the first trimester of pregnancy [13]. An increased mRNA expression of iNOS has been registered in cervical tissue at term labour compared to the non-pregnant state [11]. A newly published study has shown that the nitric oxide metabolite levels in cervical fluid in women going beyond term was 4,5 times (p < 0.001) lower that in women delivering at term [20].

To our knowledge, there are no studies so far investigating NOS expression and localization in the in preterm cervical ripening.

The purpose of our study was to investigate the mRNA expression and protein localization of the three isomers of NOS in human cervix during preterm birth, and to compare it with the conditions at term. We therefore estimated the mRNA expressions of bNOS, eNOS and iNOS, using Real-Time Multiplex RT-PCR and identified and localized synthases using immunohistochemistry.

## Methods

### Material

A total of 42 women undergoing singleton pregnancies were included in the present study. The two preterm groups included 15 women in preterm labour, (PTL) and 5 in preterm not in labour (PTnotL), delivered by caesarean section before the onset of labour. Premature delivery was defined as delivery before the 37^th ^week of gestation. Each preterm group was compared with the same group at term: 14 women in term labour (TL) and 8 in term not in labour caesarean section (TnotL). There were no significant differences regarding maternal age, parity and previous preterm births between the four groups.

The labour groups were in active labour and demonstrated a ripe cervix dilated > 4 cm. In all patients delivered by caesarean section the assessment of cervical dilatation was established immediately before surgery, by the same obstetrician (SAT) through vaginal digital examination. The women in preterm labour were either delivered vaginally, or by emergency caesarean section due to malpresentation. The women in term labour were also either delivered vaginally or by emergency caesarean due to threatening foetal asphyxia. Women not in labour had unripe cervices (Bishop score <5p) and were delivered by caesarean section before the onset of labour. The preterm indications were suspected ablatio or intra-uterine growth retardation and the term indications were breech presentation, humanitarian or disproportion. The patients with pre-eclampsia, diabetes or other systemic diseases were excluded from the study. In all women clinical signs of infection were absent, during parturition as well as during the postpartal period.

This study was performed after receiving the approval of the local Ethics Committee of the Karolinska Institute (Ref. No. 97-089) and the informed consent of each subject.

Immediately following parturition, a biopsy from the anterior cervical lip was taken transvaginally (at the 12 o'clock position) with scissors and tweezers. Our group has since 25 years applied this technique to get samples including squamous and cylindrical epithelium, vessels, glands and ECM. These samples were immediately frozen in liquid nitrogen and stored thereafter at -70°C.

Due to the limited amount of tissue from each woman, all of the different analyses could not be performed on each sample.

### Determination of mRNA

#### RNA extraction

Using Trizol reagent total RNA was extracted from the frozen biopsies [21,22].

#### DNAse treatment

DNA contamination was minimized by DNAse-I (Cat. # 2222, Ambion Inc., Austin, TX, USA) treatment.

Concentrations of total RNA were determined by measurements of the OD_260 _using a spectrophotometer (DU64^®^, Beckman, Palo Alto, CA, USA). Only samples with an OD_260_/OD_280 _ratio between 1.75 and 2.1 were used for RT-PCR.

### Real-Time Multiplex RT-PCR

The cDNA sequences of human iNOS, eNOS, and bNOS were obtained from the Entrez Nucleotide Database ; database accession numbers are shown in table 1). Specific primers and Taqman^® ^probes were designed using Primer Express^® ^software (AppliedBiosystems; primers and probes are shown in table 1). Alignment to other known human genes was excluded by running a BLAST .

**Table 1:** Description of primers and probes used for Real-time RT-PCR. Accession number (Entrez Nucleotide Database), sequences of primers and probes for human iNOS, eNOS, and bNOS; optimal primer and probe concentrations; number of PCR-cycles.

**Target**	**Accession #**	**Type**	**Sequence**	**Concentration (nM)**	**N cycles**
**iNOS**	XM_034166	**FWD**	TGGATGCAACCCCATTGTC	300	60
		**REV**	CCCGCTGCCCCAGTTT	50	
		**Probe**	6FAM-TCCCCACGGCATGTGAGGATCA-TAMRA	200	

**eNOS**	AF400594	**FWD**	CGGCATCACCAGGAAGAAGA	300	50
		**REV**	CATGAGCGAGGCGGAGAT	300	
		**Probe**	6FAM-TTCACGGCGTTGGCCACTTCTTTAAA-TAMRA	200	

**bNOS**	NM_000620	**FWD**	GGATCACATGTTCGGTGTTCAG	300	50
		**REV**	CCCAACTTTGCGCTTGAAGA	900	
		**Probe**	6FAM-AAATCCAGCCCAATGTCATTTCTGTTCG-TAMRA	200	

The experiments were carried out using the ABI Prism^® ^7700 Sequence Detection system (TaqMan^®^) from AppliedBiosystems (Foster City, CA, USA).

The conditions for multiplex RT-PCR were optimised and performed as follows (all products from AppliedBiosystems Inc., Foster City, CA, USA) with the expression of ribosomal RNA serving as internal standard:

1× TaqMan Buffer, 5.5 mM MgCl_2_, 300 μM dATP, 300 μM dCTP, 300 μM dGTP, 600 μM dUTP, 1.25 U AmpliTaq Gold DNA Polymerase (TaqMan PCR Core Reagent Kit, Part # N808-0228), 25 nM ribosomal RNA FWD-Primer, REV-Primer, and probe (Part # 4308329), 20 U RNAse inhibitor (Part # N808-0119), 12.5 U MultiScribe Reverse Transcriptase (Part # 4311235). The target RNA primer and probe concentrations used are displayed in table 1.

Reverse transcription was performed at 48°C (30 min) followed by thermal activation of the DNA polymerase (95°C, 10 min). PCR was carried out running 50–60 cycles (denaturing: 95°C [15 s]; annealing: 60°C [1 min]).

The threshold cycles (C_T_), at which an increase in reporter fluorescence above the baseline signal could first be detected, were determined.

The relative amounts of target RNA were normalized to the amounts of ribosomal RNA using the Comparative (ΔΔC_T_) Method according to the TaqMan User Bulletin #2.

### Immunohistochemistry

Due to small size of the biopsies, immunohistochemistry analyses were carried out in only two samples in each study group.

Frozen sections, 8 μm thick, were mounted onto gel chromatin-coated glass slides. The sections were fixed in 2% paraformaldehide in phosphate-buffered saline (PBS), pH 7.4, during 20 minutes, washed in PBS, and then air-dried and stored at -20° until analysis. Tissue sections were stained using the avidin-biotinylated (ABC)-peroxidase complex method. When analysed they were rewashed in PBS for three times five minutes. The endogenous peroxidase activity was eliminated by pre-treatment with 0.3% hydrogen peroxide in methanol for 30 minutes followed by washing in PBS/BSA (0,05%).

The slides were incubated over night with the primary mouse monoclonal antibodies for iNOS, eNOS and bNOS 5 ug/ml (Transduction Laboratories, Lexington, Kx) in PBS containing 0,05% bovine serum albumin. Subsequently the sections were directly incubated with the secondary antibody (horse anti-mouse) diluted in blocking sera. The sections were washed with PBS/BSA, incubated with ABC-complex for 45 minutes, and then rewashed in PBS/BSA. The reaction was developed using the DAB-kit (diaminobenzidine) from Vector (Burlingame, CA, USA). The slides were then rinsed in distilled water. Counterstaining was performed with 10% Mayer's Haematoxylin for 3–4 minutes, thereafter the sections were washed in water. For control, sections were stained as above, omitting the primary antibody. The slides were finally mounted with glycerolgelatin. For all immunohistochemical examinations the immunoreactivity was checked in the squamous epithelium, the glandular epithelium, the stroma and the vascular endothelium. The slides were classified by three independent observers using light-microscopy. Staining was localized but no attempt was made to quantify staining in the different groups.

### Statistical analysis

Outliers were removed according to Chauvenet's criterion. Data were then checked for normality. One-Way ANOVA followed by multiple pairwise comparisons (Turkey) was performed to determine differences among the groups when the data was normally distributed; when data was not normally distributed, Kruskal-Wallis One-Way ANOVA followed by multiple pairwise comparisons (Dunn's) was used. For comparison of only 2 groups, t-test or Mann-Whitney U-test were used as appropriate. A p-value of less than 0.05 was considered to indicate statistical significance.

## Results

### Determination of mRNA

Real-Time Multiplex RT-PCR confirmed the presence of mRNA for each of the three NOS isoforms within all biopsies.

#### iNOS

In general, iNOS mRNA levels were low in most of our cervical samples.

**Figure 1:**
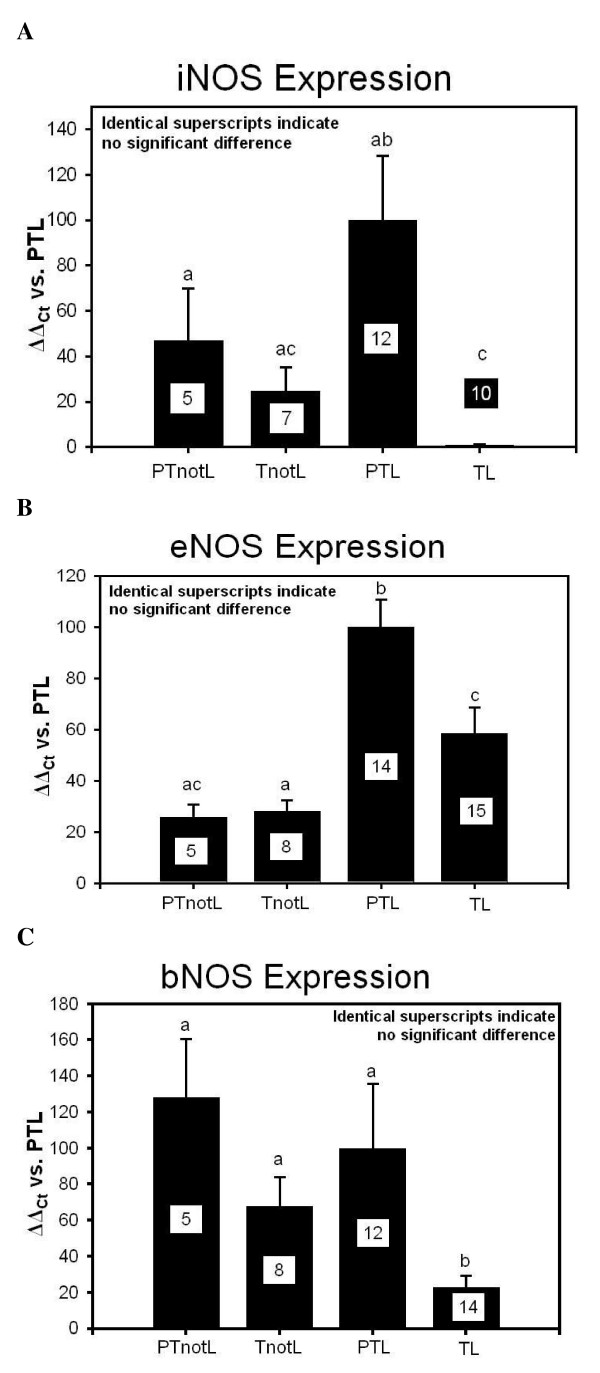
**Expression of mRNA of NOS isomers in the cervical tissue**. Expression of NOS mRNA normalized to the 'Preterm labour' group. Common superscripts indicate no significant differences. The number of patients analysed in each group are marked in each bar in the bar chart. **The groups are**: Preterm labour (**PTL**), Term labour (**TL**), Preterm not in labour, (**PTnotL**), Term not in labour, (**TnotL**). **1A:Expression of iNOS mRNA: **Patients who delivered preterm had higher iNOS mRNA levels compared to those who delivered at term. This relationship reached significance for those who were in labour (p < 0,002). **1B: Expression of eNOS mRNA: **Significantly higher levels of eNOS mRNA were registered in women with preterm labour compared to term labour (p = 0,009). Women not in labour at preterm and at term had significantly lower eNOS mRNA levels compared to preterm labour (p < 0,001) or term labor (p = 0,048) respectively. **1C: Expression of bNOS mRNA : **Women who delivered preterm had generally higher bNOS mRNA levels compared to those who delivered at term, reaching significance in the labour group (p = 0,006). The lowest values were seen in those who were in labour at term. Women who were delivered by caesarean section appeared to have higher bNOS mRNA levels than those who were in labour, reaching significance in the term groups (p = 0.007).

Patients who delivered preterm had higher iNOS mRNA levels compared to those who delivered at term. This relationship reached significance for the patients in labour (p = 0.002) (Fig. 1)

#### eNOS

Significantly higher eNOS mRNA levels were registered in the preterm labour group when compared with the term labour group (p = 0.009).

The two groups of patients with unripe cervices, thus the preterm and term not in labour groups, had significantly lower eNOS mRNA levels compared to the respective labouring groups (PTnotL vs. PTL [mean ± SEM]: 25.9 ± 4.8 vs. 100.0 ± 10.8, p < 0.001; TnotL vs. TL [mean ± SEM]: 28.1 ± 4.5 vs. 58.5 ± 5.2, p = 0.48) (Fig. 1).

#### bNOS

The lowest bNOS mRNA levels were seen in women at term labour (TL [mean ± SEM]: 23.1 ± 6.4). Women who delivered preterm generally had higher bNOS mRNA levels compared to those delivered at term, reaching significance in the group of patients in labour (PTL vs. TL [mean ± SEM]: 128.2 ± 32.6 vs. 23.1 ± 6.4, p = 0.006) (Fig. 1).

Also women who were delivered by caesarean section appeared to have higher bNOS mRNA levels than those who were in labour, reaching significance in the term groups (TnotL vs. TL [mean ± SEM]: 67.8 ± 16.4 vs. 23.1 ± 6.4, p = 0.007) (Fig. 1).

### Immunohistochemistry

As earlier mentioned, only two samples were examined from each group due to shortness of the material. All three isoenzymes were identified and localized in all samples. The negative control sections for each of the synthases showed no staining. Although, it was not possible to do quantification of immunoreactivity, but there are some observations we want to focus upon.

**Figure 2:**
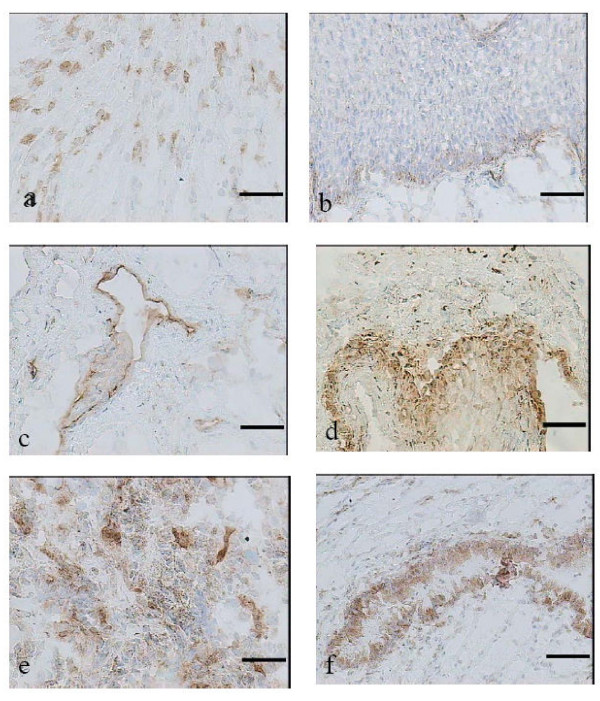
**Immunohistochemical localization of nitric oxide synthases in human cervix**. **(a) **inducible nitric oxide (iNOS) localized to the stroma in preterm labour **(b) **iNOS localized to the squamous epithelium in preterm not in labour patient. In each of the biopsies iNOS was localized in the stroma and the epithelium. **(c) **Endothelial nitric oxide (eNOS) localized to the vascular endothelium in all biopsies. This is collected from a woman in preterm labour. Neuronal nitric oxide (bNOS) had a distinct staining and was generally localized to the: **(d) **basal membrane of the squamous epithelium, picture from a women in preterm labour, **(e) **the stroma, biopsy from at term in labour patient, **(f) **the cervical glands, this sample from at term in labour patient. Original magnification × 200, scale bar 50 μm.

**iNOS **was found in the epithelium in many of the samples and some iNOS was found in the stroma in all study groups (Fig. 2). There was only one sample showing iNOS in the vascular endothelium or in the glandular cells.

**eNOS **was constitutively and prominently expressed in the vascular endothelium in all groups without any visual differences between labouring and non-labouring groups (Fig. 2).

**bNOS **showed the most impressive staining of the three isoenzymes, localized to the stroma, the glandular epithelium and the basal membrane of the squamous epithelium. The stromal localization was predominant. The visual impression was that bNOS was more expressed in the labouring groups (Fig. 2).

## Discussion

Despite intense research over the years, the complex, but well coordinated mechanisms that control the onset and maintenance of labour remains unrevealed [1,23-25]. Until now the research on preterm parturition has mainly been focused upon the myometrial activities and on potential inhibitors of preterm labour.

To our knowledge, this is the first study investigating NOS isomers expression in preterm cervix.

Our hypothesis is that a cervical remodelling occurs at preterm delivery as well as at term. It seems relevant to believe that a woman going into preterm labour starts from a relatively more unripe cervical status than a woman going into labour near or at term [26]. To achieve this more extensive remodelling from a very unripe cervical state, higher level of iNOS, eNOS and bNOS mRNA at preterm would be relevant. Here we analyse biopsies from women with preterm parturition without any signs of infection. We identified the presence of all three NOS isomers mRNA in preterm and term cervix before and after onset of labour. The most prominent finding was higher mRNA levels in preterm labour compared to term labour.

The eNOS mRNA level was significantly higher for the preterm group in labour compared to all other groups. The eNOS mRNA levels in the women not in labour, thus with unripe cervices, were significantly lower compared to those in labour irrespective of gestational age. These results may indicate a role for eNOS in the very final cervical ripening both preterm and term.

The iNOS mRNA levels were generally low in all groups, exhibiting the lowest value in the term labour group and the highest value in the preterm labour group. We have only found two earlier studies analysing iNOS and eNOS mRNA levels in human pregnant cervix [11,27]. Tschugguel et al. noted higher iNOS mRNA levels in the postpartum group compared to non-pregnant controls while Yoshida et al [27] identified both iNOS and eNOS mRNA in the first trimester cervix. In the study by Ledingham et al. labouring and non labouring patients were compared at term pregnancy and the protein expression was examined by Western blot and immunohistochemistry without any differences of the three synthases, iNOS, eNOS and bNOS, being found [13]. These different results reveal the complex story of human labour as compared to the more uniform process described in other species [7,28].

Our results showed bNOS mRNA levels to be higher in the preterm compared to the term groups. Women in preterm labour had significantly higher bNOS mRNA levels compared to the same group at term. Interestingly, when comparing labour and not in labour groups in preterm and term patients respectively, we found that the groups not in labour had higher bNOS mRNA expression levels. This is in contrast to the study of Bao et al. where an upward trend of bNOS mRNA by RT-PCR during labour compared to not in labour was noticed.

The immunoreactivity of the NO synthases had different localization. iNOS was identified in the stroma and in the epithelium. This is in line with earlier findings [11,14,27]. On the other hand we only found iNOS localised to the vascular endothelium in very few sections whereas Ledingham et al. showed iNOS protein localized to the vascular endothelium more generally [13]. The differences observed may be due to the fact that Ledingham et al. used paraffin-embedded sections of cervical tissue while we in our study, just as Tschugguel et el. and Yoshida et al. used cryosections. The eNOS specific staining was localized to the endothelium in all groups, which is supported by earlier investigations [11,13,14,27].

bNOS showed the most impressive immunoreactivity in our study. It was localised to the stroma, the glandular epithelium and to the basal membrane of the squamous epithelium. These observations agree with earlier findings by Bao et al. namely studies on cervical tissue from nonpregnant and pregnant women [12]. Our data differs from that of Ledingham et al. who could not identify bNOS in the cervical glands [13]. Tschugguel et al could not identify any bNOS at all in the cervical tissue, but this may be due to the fact that they used a polyclonal antibody, while we used a monoclonal antibody [11].

A possible concern regarding the immunohistochemistry analysis is that the interpretation of the localization of the different NO synthases might have been more reliable if a greater number of biopsies had been studied. Due to a shortage of material only two biopsies from each of the four groups were analysed in this study.

In this study we endeavoured to include women without any clinical signs of infection, neither during labour/delivery nor during the postpartum period. However, it is well known that a systemic or intrauterine infection can cause a preterm delivery and labour [29]. In those cases studies often shows increased cytokine response and up-regulation of the synthesis of cytokines compared to term non infected labour. In Sweden only around 25 % – at a maximum – of the preterm births seems to have an infectious genesis, but in other parts of the world, infection is estimated to be associated with preterm labour/delivery in as much as 40% of the cases [30]. Infection as causal to preterm labour and delivery is more frequent the lower the gestational age as well as in preterm premature rupture of membranes [31].

A new enigma based on earlier findings by our group and others is that a significant up-regulation of inflammatory parameters, cytokines, chemokines and MMPs are seen also in normal vaginal labour at term where no infection is present [1,11,32-35].

It is well known that cytokines, chemokines, different MMPs, prostaglandins, Cyclooxygenas (COX) and NO are inflammatory mediators. The extensive infiltration of immune cells as leukocytes, neutrophils and macrophages into the cervical stroma produce pro-inflammatory cytokines and collagenases that promote and accelerate the degradation of the ECM. The actions of prostaglandins and nitric oxide are activated by these cascade reactions and leads to the fully ripened cervix and induction of labour [5,19,22,32,33,36-38].

Nitric oxide donors are potent cervical ripeners and the up-regulation of NO by the proinflammatory cytokines is proposed to represent the final common pathway of cervical ripening [7,10,15,18-20]. NO directly stimulates COX-II and together with the increase of the proinflammatory cytokines IL-1, TNF-alpha and IL-8 results is increased prostaglandin production.

The increased amount of macrophages is associated with enhanced iNOS activity [13]. A strong expression of bNOS protein and mRNA in the cervical stroma at term compared to that of non-labouring or non-pregnant state is also seen [12,39].

This study revealed a frequent occurrence of bNOS in the cervix during pregnancy and labour, which is in agreement with our earlier findings that the cervix, in contrast to the uterus, is well innervated during pregnancy and labour. The immune-related cervical ripening process may be regulated by neural signalling by different neurotransmitter [40,41]. Yellon et al. also raised the question why antibiotic treatment has failed to either prolong labour or reduce the rate of preterm birth. Taking into account the theory that the cervical remodelling consists of inflammatory immune cell activation it is logical that antibiotics have no effect, as they do not affect immune cell trafficking. It thus seems plausible that the rapid final cervical ripening is not related to an exogenous infection but is instead a part of a physiological inflammatory reaction. Such a process has been registered in the endometrium during menstruation as well as in the uterus at involution postpartum [25,42,43].

## Conclusion

All the three isomers of NOS were identified in human cervix at preterm. The mRNA expression of the NOS isomers was significantly higher in the preterm group in labour compared to the women at term labour. Furthermore, the eNOS mRNA level was significantly higher during labour compared to not in labour irrespective of gestational age, which may indicates a potential role of eNOS in the very final cervical ripening.

In conclusion, our results show differences in the preterm and term mRNA expression of NOS. Further studies on preterm patients with infections are motivated, to elucidate the possible differences in NO expression in PTL with infectious genesis compared to idiopathic PTL.

## Authors' contributions

SAT have selected and recruited the patients, collected all the biopsies, participated in the design of study, did a part of mRNA extraction, drafted the manuscript. HM carried out Real-time RT-PCR analyses, performed the statistical analysis, was involved in analysis and interpretation of mRNA data. AK participated in analysis and interpretation of data, drafted the manuscript. REG participated in the design of the study, discussion of the results, drafting the manuscript. BB participated in the design of the study, mRNA extraction, discussion of the results, drafting the manuscript. AM was involved in design of the study, mRNA extraction, discussion of the results and revising of the manuscript. GEO participated in the design of the study, analysis and discussion of the results, drafting and critical revising of the manuscript. All authors read and approved the final manuscript.
